# Antioxidant Effects of Aqueous Bidens pilosa in Fructose-Fed Rats

**DOI:** 10.7759/cureus.97222

**Published:** 2025-11-19

**Authors:** S Umamaheswara Raju, S Shanmugapriyan, T Chakradhar, S Jaikumar, Suresh Babu Sayana, Natarajan Muninathan

**Affiliations:** 1 Department of Pharmacology, Meenakshi Academy of Higher Education and Research, Chennai, IND; 2 Department of Pharmacology, Meenakshi Medical College Hospital and Research Institute, Kanchipuram, IND; 3 Department of Pharmacology, Government Medical College, Maheshwaram, IND; 4 Department of Pharmacology, Panimalar Medical College Hospital and Research Institute, Chennai, IND; 5 Department of Pharmacology, Government Medical College and General Hospital, Chennai, IND; 6 Central Research Laboratory, Meenakshi Medical College Hospital and Research Institute, Kanchipuram, IND

**Keywords:** antioxidant activity, bidens pilosa, chronic fructose feeding, metabolic syndrome, oxidative stress, phytochemicals

## Abstract

This narrative review synthesizes preclinical evidence on the antioxidant potential of simple water extracts from the aerial parts of *Bidens pilosa* in rodent models exposed to chronic fructose feeding. It examines study designs, aqueous extract preparation, and outcome measures, with emphasis on redox and inflammatory endpoints. Across reports, aqueous *B. pilosa* reduces lipid peroxidation, restores endogenous antioxidant defenses (superoxide dismutase (SOD), catalase (CAT), glutathione peroxidase (GPx), and reduced glutathione (GSH)), and improves histopathology in organs affected by diet-induced stress. These effects coincide with favorable changes in metabolic and inflammatory markers, including attenuation of nuclear factor kappa-light-chain-enhancer of activated B cells (NF-κB) and tumor necrosis factor-alpha (TNF-α) signaling, consistent with broad cytoprotective activity in the liver and kidney. However, the coherence of the evidence is limited by methodological heterogeneity, including variable extract composition and dosing, uneven biomarker panels, and a limited mechanistic depth for aqueous fractions compared with organic extracts. Few studies link quantified phytochemistry with in vivo exposure-response, and pharmacokinetic data remain scarce. Clinical translation is currently absent.

Despite these gaps, the translational implications are clear. The fructose-fed model provides a pragmatic, human-relevant platform for screening antioxidant interventions. Focusing on aqueous extracts reflects real-world preparation practices and supports efforts to develop standardized, quality-assured products. Priorities include (i) consistent phytochemical profiling with batch-release markers, (ii) exposure-effect study designs integrating oxidative and inflammatory endpoints, (iii) formulation strategies to enhance bioavailability, and (iv) phased human studies in metabolic-risk cohorts incorporating safety, pharmacokinetic, and target-engagement assessments. In summary, aqueous *B. pilosa* shows reproducible antioxidant and cytoprotective activity under fructose-induced stress, supporting a structured path toward standardization and early clinical evaluation.

## Introduction and background

Ethnopharmacological significance of *Bidens pilosa*

**Figure 1:**
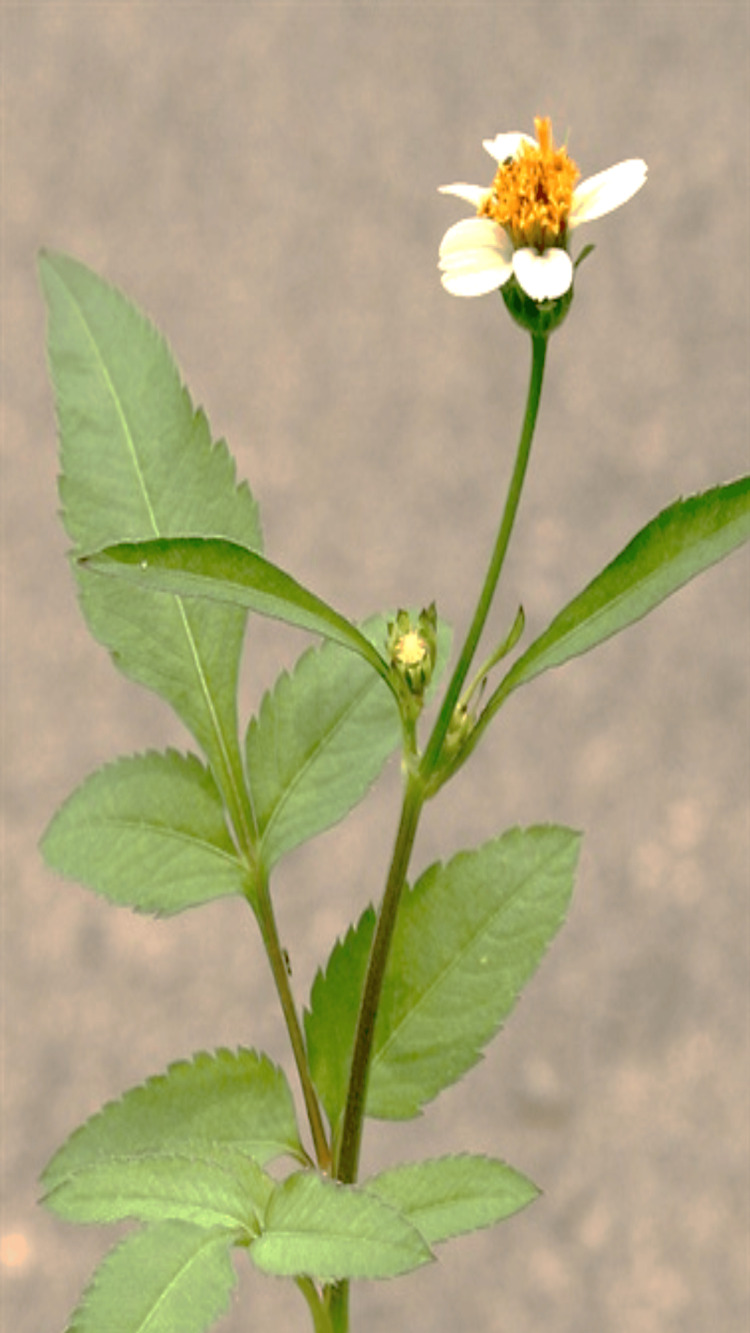
Bidens pilosa plant (aerial parts) Note: This picture was originally photographed in the Kothagudem area of Telangana, India.

Medicinal plants have historically played a pivotal role in healthcare across civilizations, offering a diverse array of bioactive compounds with therapeutic potential. Among these, *B. pilosa* (family: Asteraceae), commonly known as Spanish needle or blackjack, is widely recognized for its traditional medicinal and nutritional uses. In many parts of South America, Africa, and Asia, people use the aerial parts of this plant, such as its leaves and stems, to prepare teas, decoctions, and infusions to treat conditions such as fever, wounds, inflammation, diabetes, and digestive issues. Alongside its healing role, the plant is also part of everyday diets, especially in areas with limited resources. The young leaves are often added to soups, stews, or salads, which are packed with important nutrients like calcium, magnesium, zinc, and dietary fiber, making the plant valuable both as a food and a natural remedy [1,2] (Figure 1).

Botanically, *B. pilosa* is a fast-growing herb with serrated pinnate leaves and barbed seeds that facilitate natural dissemination. It thrives in varied habitats, from roadsides and cultivated fields to forest fringes, and is now naturalized in over 130 countries, particularly in tropical and subtropical zones [3]. From a pharmacological point of view, *B. pilosa* contains a rich mix of natural compounds that give it its healing potential. These include flavonoids like quercetin and luteolin, phenolic acids such as chlorogenic and caffeic acids, and polyacetylenes like cytopiloyne. Together, these substances act as powerful antioxidants and also show anti-inflammatory, liver-protective, and antidiabetic effects. What makes the plant especially interesting is that its water-based (aqueous) extracts, similar to the way it is traditionally prepared, have shown promising results in lab studies, particularly in reducing oxidative stress and improving metabolic health in animal models. Because it grows easily, is packed with nutrients, and holds multiple health benefits, *B. pilosa* stands out as a strong natural option for antioxidant therapy, especially in communities where access to conventional medicines is limited and chronic metabolic diseases are rising [4]. This review looks closely at the antioxidant effects of aqueous-based extracts made from the aerial parts of *B. pilosa*, especially in the context of oxidative stress caused by long-term fructose intake in animal studies. It brings together findings from existing preclinical research to identify the main active compounds, understand how they affect the body, and point out where more studies are needed. The goal is to support future research on how plant-based remedies like *B. pilosa* might help manage oxidative stress linked to metabolic diseases.

## Review

Overview of oxidative stress and reactive oxygen species (ROS) in chronic disease pathogenesis

Oxidative stress plays a key role in the development and worsening of many long-term health problems, like metabolic syndrome, diabetes, heart disease, neurodegenerative conditions, and even some types of cancer [5-7]. It happens when there is an imbalance in the body when harmful molecules called reactive oxygen species (ROS) are produced in excess, and the body’s natural antioxidant defenses cannot keep up. Normally, small amounts of ROS, like superoxide (O₂⁻), hydrogen peroxide (H₂O₂), and hydroxyl radicals (•OH), help the body by supporting cell communication and immunity. But when these molecules build up too much or stick around too long, they start damaging vital parts of the cell, such as fats, proteins, and DNA. This damage can then set off inflammation, interfere with how the mitochondria (our cells' energy producers) function, and even lead to cell death [8,9].

In metabolic diseases, particularly those associated with high-caloric diets rich in fructose or saturated fats, chronic oxidative stress has been recognized as a key molecular driver. Excessive fructose consumption alters mitochondrial metabolism and promotes de novo lipogenesis, which increases ROS production and reduces the efficiency of endogenous antioxidant systems such as superoxide dismutase (SOD), catalase (CAT), and glutathione peroxidase (GPx). This oxidative burden contributes to insulin resistance, endothelial dysfunction, hepatic steatosis, and low-grade systemic inflammation, signs of metabolic syndrome [9].

Furthermore, oxidative stress interacts synergistically with other pathological pathways, including endoplasmic reticulum stress, altered redox signaling, and inflammatory cytokine cascades (e.g., tumor necrosis factor-alpha (TNF-α), interleukin-6 (IL-6), enhancing tissue damage and disease progression. Persistent oxidative insults can also compromise genomic stability and impair cellular repair mechanisms, accelerating the transition from subclinical metabolic disturbance to overt chronic disease [8,9].

*B. pilosa* in chronic fructose-fed models of oxidative stress

Choosing *B. pilosa* as the herbal treatment and using a chronic fructose-rich diet as the experimental setup make sense because both are closely tied to issues of oxidative stress and metabolic imbalance. In today’s world, diets high in fructose, mostly from sugary drinks and processed foods, have become alarmingly common. Over time, consistently consuming large amounts of fructose can disrupt how the body handles sugar and fats. Studies in animals have shown that such diets can lead to health problems that resemble human metabolic syndrome, including high blood sugar, abnormal cholesterol levels, fatty liver, insulin resistance, and widespread oxidative stress throughout the body [10]. As such, fructose-fed rodent models serve as a robust and reproducible platform for investigating the pathophysiological consequences of dietary excess and evaluating potential therapeutic agents that target oxidative pathways [11]. Against this backdrop, *B. pilosa* emerges as a key candidate for investigation due to its rich ethnomedicinal history and well-documented pharmacological potential. The aerial parts of the plant, commonly used in traditional remedies, are particularly abundant in polyphenols, flavonoids, and polyacetylenes, phytoconstituents known for their potent antioxidant and anti-inflammatory activities [4]. Multiple animal studies have shown that *B. pilosa* can help reduce oxidative stress levels, balance blood sugar and cholesterol, and protect the liver from damage. It also shows promise in controlling inflammation. What is especially useful is that the water-based extract, prepared in the same way it is traditionally used, has been the focus of many of these studies. This makes the results more meaningful and possibly more relevant when thinking about how the plant might work in humans [9,10,12].

Looking at *B. pilosa* in the setting of chronic fructose-driven oxidative stress helps explore not just how it might treat damage, but also how it could prevent it. This dual view sheds light on how plant-based remedies can play a role in managing modern lifestyle diseases. It also opens the door to using affordable, natural antioxidants, something that could make a real difference in places where access to expensive drugs is limited, but metabolic illnesses are growing fast. Using fructose-fed models, which mimic real disease processes, alongside a plant with strong traditional roots like *B. pilosa*, gives us a practical and meaningful way to assess the value of herbal therapies in fighting oxidative stress and metabolic problems [13].

While *B. pilosa* has been traditionally used for its medicinal properties and several preclinical studies have reported its beneficial effects in metabolic disorders, there is a notable lack of focused investigations evaluating its antioxidant mechanisms in the context of fructose-induced oxidative stress. Existing studies involving *B. pilosa* in chronic fructose-fed rat models have primarily assessed cardiovascular, metabolic, and histological outcomes, yet few have quantified key oxidative stress biomarkers such as ROS, lipid peroxidation (MDA), or antioxidant enzyme activities (e.g., SOD, CAT, GPx). Moreover, aqueous extracts, despite being the most relevant to traditional usage, remain underexplored in mechanistic terms compared to organic solvent extracts. This gap in mechanistic clarity limits the translational understanding of *B. pilosa’s* antioxidant potential and available evidence to guide future research directions [1,3,4,8-11,13].

Phytochemical composition of *B. pilosa*

*B. pilosa* is a phytochemically rich medicinal herb known for its broad spectrum of bioactive components that contribute to its potent antioxidant and therapeutic properties. Among these, flavonoids, polyacetylenes, phenolic acids, aurones, and coumarins have received the most scientific attention due to their well-established roles in oxidative stress modulation [4]. Flavonoids are particularly abundant in *B. pilosa*, with quercetin and its glycosidic derivatives being the most extensively studied. Quercetin, a highly active flavonol, is capable of directly neutralizing ROS, chelating transition metals, and upregulating endogenous antioxidant enzymes such as SOD and GPx [14]. Flavonoid glycosides like quercetin-3-O-rhamnoside and luteolin-based compounds are also present in aqueous extracts and have demonstrated high solubility and biological efficacy, particularly in scavenging free radicals and preventing lipid peroxidation. In addition to flavonoids, *B. pilosa* contains significant quantities of polyacetylenes, especially phenylheptatriyne derivatives such as cytopiloyne, which possess both antioxidant and immunomodulatory activity, likely mediated by the inhibition of ROS-generating enzymes and suppression of pro-inflammatory cytokines [4,14-16].

Phenolic acids such as chlorogenic acid, caffeic acid, and ferulic acid are also key contributors to the plant’s antioxidant profile. These compounds have been shown to inhibit oxidative damage by quenching ROS, preventing lipid peroxidation, and enhancing intracellular levels of GSH. They may also activate the Nrf2/ARE pathway, a master regulator of cellular antioxidant responses [17]. Although present in smaller quantities, aurones and coumarins found in *B. pilosa* further enhance its therapeutic profile by inhibiting enzymes like xanthine oxidase and lipoxygenase and modulating the expression of inflammatory mediators such as TNF-α and IL-6. Importantly, the antioxidant efficacy of these phytochemicals is closely related to their chemical structure, and structure-activity relationship (SAR) studies have identified several key features that enhance bioactivity. For example, flavonoids with ortho-dihydroxyl groups on their rings tend to have stronger radical scavenging ability. Their conjugated double bonds and flat, planar structures also help by allowing better electron delocalization, which stabilizes antioxidant intermediates. The way these molecules are glycosylated affects how easily they dissolve and how well they are absorbed in the body, though too much glycosylation can limit their ability to interact directly with free radicals. In the same way, the lipophilic character of polyacetylenes helps them embed within cell membranes, offering protection against oxidative damage at the lipid bilayer level. Altogether, these structural features and the range of active compounds in *B. pilosa* help explain its strong antioxidant potential, especially in the context of oxidative stress triggered by chronic fructose consumption [4,15,18-21].

Experimental models and methodological approaches: chronic fructose-fed rat model

The chronic fructose-fed rat model is now widely used to study how diet-driven oxidative stress and metabolic imbalances develop over time. In this setup, rats are given fructose, usually between 10% and 20% concentration in their drinking water or food for several weeks. This prolonged exposure triggers a series of metabolic disturbances that mirror many aspects of human metabolic syndrome, such as hyperinsulinemia, insulin resistance, dyslipidemia, hypertension, increased visceral fat, and hepatic steatosis. A key part of this process is the excessive generation of ROS, driven by heightened mitochondrial respiration, increased NADPH oxidase activity, and elevated fructose-induced lipogenesis. Unlike glucose, fructose is mainly metabolized in the liver and bypasses several regulatory checkpoints. As a result, it provides abundant substrate for de novo lipogenesis, leading to an abnormal buildup of lipids inside cells. These changes disrupt mitochondrial function and intensify oxidative stress by promoting lipid peroxidation, depleting glutathione, and reducing the activity of essential antioxidant enzymes like SOD, catalase (CAT), and GPx [22].

Oxidative stress caused by high-fructose intake is more than just a chemical imbalance; it plays a central role in driving systemic inflammation, impairing endothelial function, and disrupting insulin signaling pathways. These underlying molecular changes make the chronic fructose-fed model highly applicable for studying human metabolic disorders, especially metabolic syndrome and non-alcoholic fatty liver disease (NAFLD). Both conditions consistently involve persistent low-grade inflammation, excessive lipid accumulation in the liver, and oxidative damage to tissues, all of which are reliably replicated in fructose-fed rodent models. Furthermore, the model’s ability to recapitulate early-stage pathophysiological processes makes it ideal for assessing the preventive and therapeutic efficacy of antioxidant interventions, including plant-based compounds such as those derived from *B. pilosa*. In this context, the chronic fructose-fed rat model provides a physiologically relevant and mechanistically informative platform for evaluating the modulation of oxidative stress pathways by natural agents and for exploring their potential to counteract diet-induced metabolic dysfunctions [23].

Methods for assessing antioxidant activities (in vitro and in vivo approaches)

A comprehensive assessment of antioxidant activity involves the integration of both in vitro and in vivo methodologies to evaluate a compound’s free radical scavenging potential, redox-modulating capacity, and biological relevance in living systems. Among in vitro techniques, the most widely employed assays include the DPPH (2,2-diphenyl-1-picrylhydrazyl) radical scavenging assay, ABTS (2,2'-azino-bis (3-ethylbenzothiazoline-6-sulfonic acid)) assay, and the ferric reducing antioxidant power (FRAP) assay. These tests are spectrophotometric and provide rapid, reproducible, and quantitative measures of a sample’s ability to neutralize free radicals or reduce oxidized intermediates. The DPPH assay evaluates hydrogen-donating ability, whereas the ABTS assay is particularly suited for both hydrophilic and lipophilic antioxidant systems. The FRAP assay reflects a compound’s capacity to reduce Fe³⁺ to Fe²⁺, serving as a proxy for electron-donating antioxidant behavior. These assays are frequently used for screening plant extracts, including those from *B. pilosa*, to determine their baseline antioxidant potential before moving to biological models [4,17,24,25].

In in vivo settings, antioxidant activity is typically assessed by measuring changes in key enzymatic and non-enzymatic defense systems following oxidative challenge. Primary biomarkers include enzymatic antioxidants such as SOD, GAT, and GPx, which are responsible for neutralizing superoxide radicals, hydrogen peroxide, and lipid hydroperoxides, respectively. Additional indicators like gamma-glutamyl transferase (GGT) serve as markers of oxidative stress-related liver injury, while 8-hydroxy-2′-deoxyguanosine (8-OHdG) is used to quantify oxidative DNA damage. These biochemical endpoints provide mechanistic insights into the capacity of natural compounds to modulate endogenous antioxidant responses under physiological or pathological oxidative stress, such as that induced by chronic fructose consumption [24].

Complementary methods, such as red blood cell (RBC) hemolysis protection assays, assess the extract’s ability to prevent oxidative membrane damage in erythrocytes, an important surrogate for lipid peroxidation. Phototoxicity assays are sometimes employed, especially when assessing compounds that might react under UV or visible light, to confirm their safety and stability when exposed to such conditions. When combined, these in vitro and in vivo approaches create a comprehensive system for evaluating how well medicinal plant extracts, like those from *B. pilosa*, perform as antioxidants. They also help draw connections between the chemical makeup of the extract and its biological effects. In the case of *B. pilosa*, using this range of tests is essential not only to support its traditional medicinal use but also to clarify its role in controlling oxidative stress linked to metabolic disorders [23,24].

In vitro antioxidant and protective effects of *B. pilosa*

A sizable body of in vitro work confirms that *B. pilosa*, especially extracts from its aerial parts, acts as a versatile redox modulator. Across standard benches, its aqueous and hydroalcoholic preparations quench DPPH and ABTS radicals, signaling both hydrogen-atom transfer and single-electron transfer pathways. These signals are not merely assay artefacts; the same extracts curb nitric oxide and hydroxyl radicals that directly injure proteins, lipids, and nucleic acids. Together, these readouts sketch a consistent profile: broad radical interception, dampened reactive species, and early protection of cellular macromolecules [3,19,26,27]. Beyond scavenging, *B. pilosa* shows reliable ferric-reducing power on FRAP, indicating a capacity to donate electrons and terminate oxidative chain reactions. This behavior tracks neatly with its phenolic-flavonoid signature caffeoyl/chlorogenic acids, quercetin, and luteolin derivatives commonly enriched in ethyl-acetate or methanolic fractions that outperform their parent extracts in side-by-side panels, in plain terms: richer phenolics, stronger reducing action. Such chemistry offers a clean mechanistic bridge between composition and function [26,28].

Cytoprotection shows up where it matters at the membrane and the genome. In lipid peroxidation systems (TBARS/MDA), *B. pilosa* attenuates oxidative breakdown of linoleate and model membranes, aligning with stabilization of erythrocytes and improved resilience in simple hepatic matrices. DNA-protection assays add another layer: fewer strand breaks and base lesions under hydroxyl radical challenge point to defense beyond free radical trapping, hinting at downstream buffering of damage cascades. In parallel, shifts in endogenous systems SOD, CAT, GPx, and GSH suggest that direct scavenging is paired with enzyme-level tuning [19,27]. The net effect is not just quieter chemistry in a cuvette, but sturdier cell surrogates under stress.

Two mechanistic threads strengthen translational plausibility. First, fraction-level chemistry matters: phenolic-dense fractions (including DiCQA-rich pools) tend to score higher on DPPH/ABTS/FRAP, reinforcing a dose structure activity relationship built on polyphenol abundance. Second, preliminary handling data indicate that these caffeoylquinic acids demonstrate tractable bioaccessibility and transport characteristics, making them realistic contributors to downstream biological effects. Put simply, the molecules that look potent in vitro are also poised to be present at the site of action. These insights align with the manuscript’s forward arc. The in vitro dossier clarifies why an aqueous aerial part extract is a defensible choice, informs dose-banding by anchoring to phenolic yield and TEAC/FRAP footprints, and motivates the selection of oxidative-stress endpoints in vivo MDA, NOx, GSH, and the SOD/CAT/GPx triad under chronic fructose load. In short, multiple constituents act in concert, the signals converge across assays, and the protective phenotype is broad. That synergy is precisely what the subsequent animal work is set to probe, with metabolic stress as the proving ground [3,19,26,27].

In vivo antioxidant and protective effects of *B. pilosa*

Long-term dosing in fructose-fed rat models continues to point in the same direction: aqueous or alcohol extracts from the aerial parts upshift endogenous defenses and pull redox tone back toward baseline. Across studies, SOD, CAT, and GPx climb, while GSH rebounds in liver and kidney changes consistent with a genuine systems-level effect rather than a single readout blip. Converging animal data in other oxidative-injury setups strengthen this signal; for example, a recent formaldehyde/formalin kidney-injury model showed a clear histological rescue with *B. pilosa* leaves (notably at 100 mg/kg), and docking pointed to flavonoids such as luteolin and isochlorogenic acids as plausible redox/anti-inflammatory levers underpinning the in vivo benefit. In short, enzyme recovery and tissue-level protection line up, suggesting the extract is not merely mopping up radicals but also stabilizing antioxidant machinery over time [18,24,29].

Parallel to enzyme shifts, downstream injury markers ease off. Work in rodent tissues has repeatedly noted lower malondialdehyde (MDA) as a lipid-peroxidation proxy, alongside drops in hepatic stress indices (e.g., GGT) and oxidized DNA adducts (8-OHdG), mirroring a broad-spectrum antioxidant effect. Mechanistically, membrane-level stabilization and conservation of red cell antioxidant capacity shown ex vivo in human erythrocytes exposed to radical generators offer a clean, biologically plausible bridge between phytochemistry and whole-organism readouts: *B. pilosa* extracts limited oxidative hemolysis and helped preserve GSH and enzyme activities, implying fortified frontline defenses that would translate in vivo. The plant’s chemical roster, flavonoids (including quercetin-type scaffolds), phenolic acids, and related constituents, maps neatly onto these effects and is well-represented in edible and medicinal preparations that reach target tissues [18,24,30,31].

Histopathology tells the same story in organs that bear the brunt of fructose-linked stress. In the kidneys, *B. pilosa* leaf extract attenuated tubular injury and pulled necrosis scores back toward normal, aligning with the enzyme and biomarker shifts above. Reports in the metabolic syndrome context extend this protective flavor to hepatocellular changes (steatosis and inflammation) and pancreatic-islet integrity, with anti-inflammatory crosstalk likely contributing; bioactives such as isookanin demonstrate NF-κB-linked dampening that pairs naturally with antioxidant gains. Complementary evidence from fructose-hypertensive rats and toxin-induced nephropathy models (e.g., tea from *B. pilosa* leaves in CCl₄ injury) broadens the applicability of these findings across oxidative stress phenotypes. Together, these in vivo strands of enzymes, biomarkers, and micro-anatomy paint a consistent picture of cytoprotection that is chemically and biologically coherent [30-33].

Antioxidant and cytoprotective effects of *B. pilosa*: mechanisms of action

The antioxidant and protective actions of *B. pilosa* operate through several interconnected mechanisms that work together to combat oxidative stress at both the cellular and molecular levels. A key mechanism is the direct neutralization of ROS and free radicals. Compounds like quercetin and luteolin (flavonoids), as well as chlorogenic and caffeic acids (phenolic acids), contribute to this effect by donating hydrogen atoms or electrons. These donations help neutralize reactive molecules such as superoxide anions, hydroxyl radicals, and peroxyl radicals. By interrupting these oxidative chain reactions, the plant helps shield essential cellular components, including lipids, proteins, and DNA, from oxidative damage [2].

In addition to direct ROS neutralization, *B. pilosa* exhibits metal-chelating properties that play a crucial role in limiting redox cycling and the generation of highly reactive hydroxyl radicals via the Fenton reaction. The plant’s polyphenolic constituents can bind transition metal ions such as Fe²⁺ and Cu²⁺, reducing their availability to catalyze lipid peroxidation and other pro-oxidant reactions. This chelation mechanism complements the plant’s ability to inhibit lipid peroxidation, as demonstrated in various in vitro and in vivo assays where administration of *B. pilosa* extract has resulted in reduced MDA levels, a key biomarker of membrane lipid oxidation [17].

A key part of *B. pilosa’s* antioxidant action involves boosting the body’s own enzymatic defense systems. Animal studies have shown that treatment with the plant’s extract leads to higher activity of crucial enzymes like SOD, CAT, and GPx. These enzymes help keep redox balance in check: SOD breaks down superoxide radicals, CAT converts hydrogen peroxide into harmless water and oxygen, and GPx reduces lipid hydroperoxides to non-toxic forms. This enhanced enzymatic activity indicates that *B. pilosa* not only offers immediate antioxidant effects through its phytochemicals but also supports and reinforces the long-term defenses against oxidative stress [2].

Furthermore, the plant modulates key inflammatory pathways that are closely intertwined with oxidative stress. Chronic oxidative insults can activate NF-κB, a transcription factor that upregulates pro-inflammatory genes including TNF-α, IL-6, and iNOS. *B. pilosa* has been shown to suppress the activation of NF-κB and downregulate the expression of its downstream mediators, thereby exerting anti-inflammatory effects that reinforce its antioxidant function. This dual antioxidant-anti-inflammatory activity is particularly beneficial in models of metabolic syndrome and non-alcoholic fatty liver disease (NAFLD), where oxidative stress and inflammation act synergistically to promote tissue damage [34].

Taken together, these mechanisms help explain the broad protective role of *B. pilosa* in conditions marked by both oxidative and inflammatory stress. The plant components act at several key points along the oxidative stress pathway, neutralizing free radicals, binding harmful metal ions, boosting antioxidant enzyme activity, and modulating inflammatory responses. This layered, multifaceted mode of action reflects not only its traditional healing role but also supports the growing preclinical evidence for its therapeutic potential (Table 1).

**Table 1:** Key antioxidant mechanisms of aqueous extract of B. pilosa in chronic fructose-fed models ROS: reactive oxygen species, MDA: malondialdehyde, SOD: superoxide dismutase, CAT: catalase, GPx: glutathione peroxidase, Fe²⁺: ferrous ion (iron in +2 oxidation state), TBARS: thiobarbituric acid reactive substances, TNF-α: tumor necrosis factor-alpha, IL-6: interleukin-6, NF-κB: nuclear factor-kappa B.

Mechanism of action	Key phytoconstituents involved	Biological markers affected	Observed outcomes in animal models
ROS removal and radical elimination [19]	Quercetin, luteolin, and chlorogenic acid	↓ ROS, ↓ MDA	Reduced lipid peroxidation and membrane damage
Upregulation of endogenous antioxidant enzymes [19]	Quercetin-3-O-rhamnoside, caffeic acid	↑ SOD, ↑ CAT, ↑ GPx	Improved redox balance; enhanced endogenous antioxidant defense
Metal chelation and inhibition of lipid peroxidation [11]	Polyacetylenes, ferulic acid, phenolic acids	↓ Fe²⁺-induced damage, ↓ TBARS	Stabilization of cellular membranes; reduced oxidative stress
Inhibition of pro-inflammatory cytokines [20]	Cytopiloyne, flavonoids	↓ TNF-α, ↓ IL-6, ↓ NF-κB activation	Reduced systemic inflammation and hepatic injury
Mitochondrial protection and energy balance [21]	Flavonoids, polyphenols	↑ Mitochondrial function, ↓ oxidative phosphorylation disruption	Restoration of hepatic energy metabolism

Safety, toxicity, and dosage considerations

The safety and toxicity profile of *B. pilosa* has been the subject of growing research interest, particularly given its widespread use in traditional medicine and emerging therapeutic potential. Acute and sub-chronic toxicity studies in animal models have generally indicated a favorable safety profile. In rodent experiments, oral administration of aqueous and methanolic extracts of *B. pilosa* at doses up to 2 mg/kg weight did not result in mortality, overt toxicity symptoms, or significant histopathological abnormalities in major organs, suggesting that the plant possesses low acute toxicity. Sub-chronic toxicity evaluations over several weeks of daily dosing have similarly shown no substantial alterations in hematological, biochemical, or hepatic indices, further supporting its safety in prolonged use. These dosing regimens have proven effective in modulating oxidative stress, improving lipid and glucose metabolism, and protecting tissue architecture in fructose-fed rat models, further affirming their pharmacological safety in controlled environments [35].

Despite these reassuring findings, some caution is warranted. Although *B. pilosa* is generally well-tolerated, isolated reports have raised concerns regarding potential herb-drug interactions, particularly when used concomitantly with anticoagulants, antihypertensives, or antidiabetic agents. This is primarily attributed to the plant’s bioactive flavonoids and polyacetylenes, which may influence cytochrome P450 enzyme activity and alter drug metabolism or pharmacodynamics. While such interactions have not been widely reported in clinical settings, they remain a theoretical concern, especially in populations using polypharmacy [4,36,37].

In terms of regulatory recognition, *B. pilosa* has not yet been granted “Generally Recognized as Safe” (GRAS) status by the US Food and Drug Administration (FDA), nor does it hold equivalent approval from major bodies like the European Medicines Agency (EMA). That said, it is listed in several national herbal pharmacopoeias, and its aerial parts are widely used as both food and traditional medicine in various countries, underscoring its long-standing cultural acceptance and anecdotal safety. Still, the lack of standardized formulations, established dosing guidelines, and robust long-term safety data calls for more thorough toxicological studies and regulatory assessments before it can be confidently integrated into evidence-based modern healthcare [4,35,38].

Eventually, *B. pilosa* appears to be safe at commonly used therapeutic doses in preclinical models, with minimal evidence of acute or chronic toxicity. However, given the potential for herb-drug interactions and the lack of formal regulatory classification, caution should be exercised in its widespread clinical use until more robust toxicological and pharmacokinetic data become available.

Gaps, limitations, and future research directions

Despite the promising preclinical evidence supporting the antioxidant and therapeutic potential of *B. pilosa*, several critical gaps and limitations must be acknowledged to contextualize its translational relevance. Foremost among these is the absence of well-designed clinical trials in human populations. To date, the majority of research has been confined to in vitro assays and rodent models, particularly in the context of diet-induced oxidative stress. While these studies provide valuable mechanistic insights, they fall short of establishing the plant’s efficacy, safety, pharmacokinetics, and tolerability in humans. Clinical investigations, especially randomized controlled trials, are urgently needed to validate their therapeutic claims, assess optimal dosing strategies, and identify possible herb-drug interactions under real-world conditions.

Another major limitation lies in the significant variability observed in the phytochemical composition of *B. pilosa*, which is influenced by factors such as geographical origin, cultivation conditions, harvesting time, and extraction method. For instance, aqueous and methanolic extracts may differ considerably in the concentrations and types of flavonoids, polyacetylenes, and phenolic acids they contain. This heterogeneity poses a challenge to reproducibility and makes it difficult to compare results across studies or develop standardized formulations. Therefore, there is a pressing need to standardize extraction protocols and quantify key bioactive constituents using validated analytical techniques such as HPLC, LC-MS, or NMR. Furthermore, very few studies have explored the bioavailability, metabolism, and pharmacodynamics of these compounds in vivo, which is essential for determining their therapeutic potential and safety in humans.

To move past current limitations and fully explore the therapeutic potential of *B. pilosa*, upcoming research should prioritize a few key directions. First, there is a need to create standardized, quality-assured extracts that maintain consistent phytochemical content. These would form a solid foundation for both preclinical testing and future clinical use. At the same time, improving how these compounds are delivered in the body is essential. Approaches like nano-formulations, liposomes, and phytosomes could enhance the bioavailability, stability, and targeted effects of *B. pilosa's* antioxidant components. Moreover, using advanced analytical tools such as genomics, transcriptomics, metabolomics, and proteomics can shed light on the deeper molecular pathways involved in how the plant combats oxidative stress and metabolic disturbances. These “omics” techniques may also uncover new biomarkers that help track both their effectiveness and safety.

Additionally, future studies should aim to explore the long-term safety of *B. pilosa*, particularly in vulnerable populations such as pregnant women, the elderly, and individuals with multiple comorbidities or polypharmacy. Comparative studies with standard antioxidants and pharmacological agents would also be valuable in positioning *B. pilosa* within the broader therapeutic landscape. Moreover, research on potential synergistic effects with other medicinal plants or dietary components could further enhance its clinical utility. Given its dual nutritional and therapeutic profile, *B. pilosa* lends itself to development as a functional food or nutraceutical, e.g., standardized aqueous extract sachets/teas, ready-to-mix powders, or fortified beverages alongside capsule formulations. Priority steps include establishing a reproducible phytochemical fingerprint for batch standardization, pairing extracts with bioavailability-enhancing excipients, and mapping dose-exposure-response in early human studies among metabolic-risk populations. These efforts should run in parallel with toxicology, palatability/acceptability testing, and claim-substantiation trials that progress from feasibility to multicenter designs. Together, this roadmap connects traditional dietary use to regulated, scalable products with measurable clinical value.

## Conclusions

*B. pilosa* is a widely available medicinal plant with deep roots in traditional medicine, which has attracted growing scientific interest due to its notable antioxidant and cytoprotective properties, especially when used in the form of aqueous extracts from its aerial parts. This review consolidates preclinical findings that support its ability to counter oxidative stress and prevent tissue injury in chronic fructose-fed rat models, which closely mimic human conditions such as metabolic syndrome and NAFLD. Its therapeutic effects are largely credited to a rich mix of flavonoids, polyacetylenes, and phenolic acids, which work by scavenging ROS, binding metal ions, inhibiting lipid peroxidation, and boosting the activity of antioxidant enzymes like SOD, CAT, and GPx. In addition, *B. pilosa* influences important inflammatory pathways such as NF-κB and TNF-α, further supporting its potential role in managing diseases driven by both oxidative and inflammatory stress.

However, a significant gap in the existing literature is the absence of robust clinical trials and the inconsistent measurement of oxidative biomarkers across studies. In addition, variability in extract composition due to geographical and methodological differences underscores the urgent need for standardization and phytochemical characterization. Future research should prioritize the development of bioavailable formulations, explore synergistic interactions with other natural agents, and employ omics technologies to unravel molecular mechanisms and identify predictive biomarkers. Subsequently, this review not only reaffirms the traditional use of *B. pilosa* as a natural antioxidant but also positions it as a promising candidate for the integrative management of metabolic and oxidative stress-related disorders. Bridging this evidence to clinical translation will require multicenter trials, formulation optimization, and rigorous toxicological profiling. Until then, *B. pilosa* remains a promising yet underutilized phytomedicine in managing oxidative stress-linked metabolic diseases.
